# ID 44 - Mapeamento de Cursos On-Line em Avaliação de Tecnologias em Saúde no Mundo: resultados preliminares

**DOI:** 10.5327/2237-9622.2025.v34s1.44

**Published:** 2025-11-25

**Authors:** Roseana Boek Carvalho, Marina Petrasi Guahnon, Muriel Primon de Barros, Bruna Marmett, Ana Paula Blankenheim, Bárbara Cristiane da Silva, Rodrigo Pereira de Almeida, Cinara Stein, Gilson Pires Dorneles, Suena Medeiros Parahiba, Maicon Falavigna

**Keywords:** cursos, educação a distância, Avaliação de Tecnologias em Saúde

## Abstract

**Introdução:**

No cenário atual, com o rápido avanço das tecnologias em saúde e a célere necessidade de avaliar sua eficácia, segurança e custo-efetividade, a formação de profissionais capacitados se tornou uma prioridade global. Cursos oferecidos no formato de ensino a distância (EaD) são essenciais para capacitar profissionais de forma ampla e acessível, independentemente de sua localização geográfica. O mapeamento de cursos dessa modalidade, focados na avaliação de tecnologias em saúde (ATS), torna-se uma iniciativa fundamental diante da crescente demanda por capacitação nesta área, a qual é peça-chave para a formulação de políticas de saúde baseadas em evidências. Justifica-se esse mapeamento pela necessidade de democratizar o acesso ao conhecimento, fortalecer a capacidade técnica em diferentes contextos geográficos e promover a tomada de decisões mais informadas e embasadas na área da saúde. O objetivo deste trabalho foi identificar a oferta de cursos EaD de alcance internacional que possam ampliar e apoiar o conhecimento em ATS disponíveis no mundo.

**Método:**

Este estudo é uma revisão de escopo baseada em uma busca exploratória sistemática em sites de agências mundiais de ATS e no site de busca Google utilizando a combinação de palavras e, realizando uma seleção dos primeiros 200 retornos. Além da busca sistemática em banco de dados (PubMed, Cochrane). Foram incluídos cursos EaD ofertados na língua inglesa, sem limite de data de oferta. Dois revisores realizaram a inclusão de acordo com a população, conceito e contexto (PCC) preestabelecido. A extração de dados incluiu: país de origem, tema, público-alvo, carga horária, tipo de modalidade EaD e taxa. Registro do protocolo: DOI 10.17605/OSF.IO/FGWXN

**Resultados:**

Até setembro de 2024 foram selecionados e identificados 50 cursos via site de busca Google, que atenderam aos critérios de inclusão e foram categorizados nos principais temas de: síntese de evidências (n=19), avaliação econômica (n=28) e diretrizes de prática clínica (n=3). O público-alvo mais frequente compreendia trabalhadores da área da saúde (56%) com carga horária de até 20 horas (80%). Entre os cursos incluídos, 46% eram autoinstrucionais e assíncronos, sendo a maioria pagos (74%). As agências patrocinadoras estavam concentradas principalmente na América do Norte (n=23) e Europa (n=22), seguido de Oceania (n=3) e Ásia (n=2).

**Conclusão:**

O presente estudo destaca a importância crescente dos cursos EaD na capacitação de profissionais em ATS. A predominância de cursos voltados para profissionais da saúde também reforça a relevância dessa capacitação para aqueles que já estão diretamente envolvidos no tema. A maioria dos cursos pagos pode limitar o acesso a essa capacitação para profissionais em países de baixa e média renda, sendo a ampliação do acesso a essas formações essencial para a construção de políticas de saúde baseadas em evidências e para o fortalecimento dos sistemas de saúde em um cenário global. As perspectivas futuras do presente estudo são a ampliação da busca em bases científicas, bem como em agências e órgãos mundiais focados em ATS, assim como a ampliação do entendimento da capacitação e da equidade no acesso ao conhecimento.

